# Reliability of a White Striping Scoring System and Description of White Striping Prevalence in Purebred Turkey Lines

**DOI:** 10.3390/ani12030254

**Published:** 2022-01-21

**Authors:** Ryley J. Vanderhout, Emily M. Leishman, Heather Hiscock, Emhimad A. Abdalla, Bayode O. Makanjuola, Jeff Mohr, Alexandra Harlander-Matauschek, Shai Barbut, Benjamin J. Wood, Christine F. Baes, Nienke van Staaveren

**Affiliations:** 1 Centre for the Genetic Improvement of Livestock, Department of Animal Biosciences, University of Guelph, Guelph, ON N1G 2W1, Canada; rvande02@uoguelph.ca (R.J.V.); eleishma@uoguelph.ca (E.M.L.); emhimad.abdalla@vit.de (E.A.A.); makanjuo@msu.edu (B.O.M.); b.j.wood@uq.edu.au (B.J.W.); nvanstaa@uoguelph.ca (N.v.S.); 2 Department of Food Science, University of Guelph, Guelph, ON N1G 2W1, Canada; hhiscock@uoguelph.ca (H.H.); sbarbut@uoguelph.ca (S.B.); 3 Hybrid Turkeys, 650 Riverbend Drive Suite C, Kitchener, ON N2K 3S2, Canada; jeff.mohr@hendrix-genetics.com; 4 The Campbell Centre for the Study of Animal Welfare, Department of Animal Biosciences, University of Guelph, Guelph, ON N1G 2W1, Canada; aharland@uoguelph.ca; 5 School of Veterinary Science, University of Queensland, Gatton, QLD 4343, Australia; 6 Institute of Genetics, Vetsuisse Faculty, University of Bern, 3001 Bern, Switzerland

**Keywords:** poultry, white striping, myopathy, pedigree line, selection, scoring system

## Abstract

**Simple Summary:**

A relatively recent issue in the turkey industry is white striping (presence of white striations on the surface of the breast fillets). This defect influences consumer acceptance and the nutritional value of the meat and, therefore, is of economic importance to the industry. This study is aimed to test the reliability of a white striping scoring system used by several observers and estimate the prevalence of this defect in modern turkeys. After a few training sessions, the scoring system was found to be moderately reliable within and between the six participating observers. We found that 88% of turkeys in the studied population had some degree of white striping, with most scores being of moderate-severe severity (Score 1 or 2). Furthermore, white striping severity was found to be associated with higher slaughter weight, breast weight, and breast meat yield. Future research is needed to evaluate the use of white striping information in turkey genetic selection programs, as a balanced approach is needed to avoid slowing gains in economically favorable traits, such as growth.

**Abstract:**

To efficiently meet consumer demands for high-quality lean meat, turkeys are selected for increased meat yield, mainly by increasing breast muscle size and growth efficiency. Over time, this has altered muscle morphology and development rates, which are believed to contribute to the prevalence of myopathies. White striping is a myopathy of economic importance which presents as varying degrees of white striations on the surface of skinless breast muscle and can negatively affect consumer acceptance at the point of sale. Breeding for improved meat quality may be a novel strategy for mitigating the development of white striping in turkey meat; however, it is crucial to have a reliable assessment tool before it can be considered as a phenotype. Six observers used a four-category scoring system (0–3) to score severity in several controlled rounds and evaluate intra- and inter-observer reliability of the scoring system. After sufficient inter-observer reliability (Kendall’s W > 0.6) was achieved, 12,321 turkey breasts, from four different purebred lines, were scored to assess prevalence of the condition and analyze its relationship with important growth traits. Overall, the prevalence of white striping (Score > 0) was approximately 88% across all genetic lines studied, with most scores being of moderate-severe severity (Score 1 or 2). As was expected, increased white striping severity was associated with higher slaughter weight, breast weight, and breast meat yield (BMY) within each genetic line. This study highlights the importance of training to improve the reliability of a scoring system for white striping in turkeys and was required to provide an updated account on white striping prevalence in modern turkeys. Furthermore, we showed that white striping is an important breast muscle myopathy in turkeys linked to heavily selected traits such as body weight and BMY. White striping should be investigated further as a novel phenotype in future domestic turkey selection through use of a balanced selection index.

## 1. Introduction

The consumption of turkey meat is increasing worldwide. It is the second most-consumed poultry meat globally, particularly in Europe and North and South America [1]. With consumer preference for smaller, deboned, or further processed products typically sold in trays covered with clear plastic film, the visual appearance of skinless portions is the most important determinant of acceptance at the point of sale [2,3,4].

Genetic selection in poultry has been geared toward increasing body weight, average daily gain, and breast muscle yield to meet consumer meat demand more efficiently [5]. This selection pressure has increased myopathies of the *Pectoralis major*, such as white striping (WS) [6,7]. From a microscopic point of view, breasts affected by WS show an increased sign of muscle cell injury thought to be related to rapid growth of the breast muscle tissue leading to ischemia and myofibril necrosis with minimal time for cell repair [8,9,10,11,12]. The necrotic muscle tissue is then infiltrated by fat and connective tissue leading to the macroscopic presence of white striations on the surface of the fillet and resulting in fillets with a higher lipid and lower protein content [8,9,13]. WS has been shown to negatively affect consumer acceptance of fresh, skinless broiler chicken meat [14,15], and one would assume the same finding is likely in turkeys. This is because consumers associate these white striations with meat from older poultry (e.g., older chickens tend to show more striations than young broiler chickens). However, today we see these white striations also in young birds [16]. WS has also been shown to affect the quality of the meat by decreasing marinade uptake, increasing cooking loss, and increasing hardness of the meat as a result of decreased functional proteins [12,17,18,19,20,21]. Consequently, larger breast fillets that are commonly used for further processing will also be negatively affected by WS.

The incidence of WS has been researched in broiler chickens, and flock prevalence between 56–87% has been reported in heavy strains [10,22,23,24]. Research on WS in turkeys is limited compared to broiler chickens, with a recent study showing a flock-level prevalence of over 60% [25]. Genetically, WS has been estimated to be moderate to highly heritable in broiler chickens [26,27,28,29], but to our knowledge, no estimates have been published for turkeys.

Although the development of WS is associated with microscopic changes to the muscle tissue [17], this myopathy is clearly observable at the macro level and can be scored without microscopes or other technologies. A four-category scoring scheme, primarily accounting for thickness of the striations, has been used to evaluate the severity of WS in broiler meat; this scheme classifies the appearance of breast meat samples as normal (no distinct white lines), moderate (small lines, <1 mm), severe (large white lines, 1–2 mm), or extreme (thick bands, >2 mm) [9,14]. This scheme has also been used in several turkey studies [25,30,31]. Zampiga et al. [32] used a similar four-category scoring scheme based on the proportion of the breast fillets covered by stripes as opposed to the thickness of the bands. 

As muscle myopathies are a rising problem in the North American meat industry [26,33,34] and consumers’ awareness of these pathologies and their concerns about animal well-being rise [35,36,37], efforts have been made by breeding companies to improve the health of the animal and the appearance and quality of meat products by reducing the occurrence of emerging muscle myopathies. To do so, it is important to find a reliable method of identifying and classifying defects such as WS. There is a lack of evaluation of the reliability of visual scoring schemes for WS with multiple observers. Accurate recording of WS is important to ensure consistency over time and between observers, especially when considering the inclusion of a trait in breeding programs [38]. The objectives of this study were to: (1) evaluate the intra- and inter-observer reliability when using a WS scoring system adapted for turkeys, (2) evaluate the prevalence and severity of WS in turkey breast muscles, and (3) evaluate associations between WS scores and production traits (i.e., breast weight, slaughter weight, and breast meat yield).

## 2. Materials and Methods

### 2.1. Animals

Adult male turkeys, 20–24 weeks old, from four purebred lines (A, B, C, and D) were processed over 44 weeks between 2018 and 2019 at a local commercial poultry processing plant as part of a larger project focusing on genomic selection in turkeys [34]. The number of birds included in this study was 12,321 with 2839, 3728, 2034, and 3720 from lines A, B, C, and D, respectively. Birds were reared under identical conditions according to Hybrid Turkeys [39] and were weighed two days prior to processing (i.e., slaughter weight) (OHAUS scale, NJ, USA, accuracy to 0.01 kg). During processing at a commercial processing plant, the birds were electrically stunned and exsanguinated. Birds were scalded, defeathered, and eviscerated before moving to the chiller for 24 h prior to deboning and collecting meat quality and breast muscle weights (OHAUS scale, NJ, USA, accuracy to 0.01 kg). Breast meat yield (BMY, %) was then calculated as a proportion of slaughter weight.

### 2.2. Scoring System

**Figure 1:**
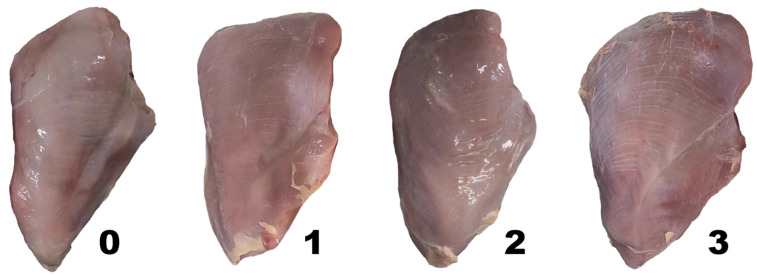
Visual scoring system used for scoring white striping severity of the Pectoralis major (fillet). Where 0 (normal) = no to minimal white striations; 1 (moderate) = thin white striations visible on the breast, most of which tended to occur at the caudal end of the fillet (bottom of pictured fillets); 2 (severe) = large white striations visible on the breast spread between the caudal end and the main body of the fillet (top of pictures fillets); 3 (extreme) = thick white striations visible on the breast covering majority of the outer surface. Pictures by Ryley J. Vanderhout.

Both *Pectoralis major* muscles were photographed (Hero 6, GoPro, San Mateo, CA, USA) approximately 24 h post mortem. Photographs were taken approximately 40 cm from the surface of the fillets using the “normal” focal length setting of the camera to minimize distortion of the image. These photographs were used to evaluate a 0–3 scoring scale for WS which was adapted from a system originally used in broiler chickens [9]. As shown in Figure 1, a score of 0 represented no to minimal white striations, Score 1 represented thin white striations visible on the breast, most of which tended to occur at the caudal end of the fillet, Score 2 represented white striations visible on the breast spread between the caudal end and the main body of the fillet, and Score 3 represented thick white striations visible on the breast covering a majority of the outer surface. If the two breasts in each photograph differed in severity, observers were instructed to record the more severe score. All observers were blind to all additionally recorded data of each image including genetic line, age at slaughter, slaughter weight, breast weight, and BMY.

### 2.3. Scoring Evaluation

The intra- and inter-observer reliability of the scoring system was assessed among six observers due to the high number of samples to be scored. One observer had previously tested the scoring system on a subset of photos to determine its feasibility, while others had no previous experience scoring WS severity. The scoring system was discussed among the observers before the first scoring session. A session consisted of all observers scoring 50 photographs in duplicate (two rounds per session). The initial subset of photographs was semi-randomly selected in that they had to give a clear visual of the breast muscle (e.g., without any damage due to trimming). Observers scored the same 50 photographs in an initial round and repeated the exercise several days later with the same set of photographs in a different randomized order. Intra-observer reliability was determined based on the results of the two rounds within one session for each observer. In contrast, inter-observer reliability was determined based on the specific round across observers. Following the first session (session 1), all observers discussed the results to align their scoring before repeating another session with the same 50 photos (session 2). A final session (session 3) was conducted after further discussion with a new subset of 50 photos that were randomly selected to reflect photographs collected under more typical processing plant conditions (e.g., excessive fat/skin obscuring the muscle, damaged breast muscle).

### 2.4. Reliability Analysis

All agreement calculations were performed using SAS^®^ statistical software, version 9.4 (SAS Institute Inc., Cary, NC, USA [40]). 

Multiple measures of agreement were calculated for intra- and inter-observer reliability to comprehensively understand the reliability of the scoring system. Intra-observer reliability was assessed by calculating exact agreement between two rounds, Spearman’s rank correlations, Fleiss–Cohen’s kappa coefficient, linear weighted kappa, and quadratic weighted kappa. Kappa statistic estimates were calculated to determine the level of agreement corrected for chance. Weighted kappa coefficients (linear and quadratic) were calculated to attribute partial credit depending on the relative extent of the disagreement between scores using Fleiss–Cohen weights [41]. Kappa values were interpreted following the classifications suggested by Landis and Koch [42] and presented in Petrie and Watson [43], where *κ* ≤ 0.20 is ‘poor’, 0.21 ≤ *κ* ≤ 0.40 is ‘fair’, 0.41 ≤ *κ* ≤ 0.60 is ‘moderate’, 0.61 ≤ *κ* ≤ 0.80 is ‘substantial’, and *κ* > 0.80 is ‘good’. It should be noted that these classifications are arbitrary [42], and the interpretation of kappa is dependent on the research field; however, a value closer to 1 indicates better agreement.

Inter-observer reliability was assessed by calculating estimates and tests of agreement among multiple observers using the %MAGREE macro (v3.8, SAS Institute Inc., Cary, NC, USA). The number of observers agreeing on each photograph was determined to calculate the percentage of cases in which 2, 3, 4, 5, or all 6 observers agreed on a score. Exact and weighted (linear and quadratic) kappa statistics were estimated [41]. Kendall’s W coefficient of concordance was estimated to test agreement of the observers’ ranking of the photographs. Finally, Gwet’s weighted agreement coefficient (AC_2_) for ordinal data was calculated to assess agreement on each score category while treating both observers and photographs as sampled rather than fixed to assess agreement of the entire populations of observers and photographs. For both Kendall’s *W* and Gwet’s agreement coefficient, the maximum value of 1 indicates perfect agreement.

### 2.5. White Striping Prevalence and Association with Production Traits

Upon completion of the three scoring sessions (six rounds), all 12,321 photos were divided among the six observers. Using R version 3.5.2 [44] and ggplot2 [45], the score frequency within each line and within the entire population was plotted. To test the influence of genetic line on WS score, a Tukey’s HSD test implemented in the stats package within R [44] was conducted on a linear model with genetic line as the only fixed effect. In addition to the WS scores, 12,282 birds had measurements for body weight two days before processing (slaughter weight), 12,190 had measures for breast weight (combined weight of both *Pectoralis major* and *minor* muscles), and 12,168 had measures for breast meat yield (BMY; breast weight as a percentage of slaughter weight). For each trait, outliers were excluded based on 3 standard deviations from the mean, and therefore, sample numbers differed slightly between the traits. Least square (LS) means of the three weight measures, for each score category, were calculated using a linear model with WS score and genetic line as fixed effects. LS means were then plotted for each genetic line. Statistical significance between the means was determined using Tukey’s HSD test implemented in the stats package within R.

## 3. Results

### 3.1. Intra-Observer Reliability

The results of the intra-observer reliability assessment in the three sessions for each observer are shown in Table 1. The percentage of exact agreement ranged between 62–78% in the first session for all observers, except Observer 1, who acknowledged having been influenced by discussion in between rounds in the first session. As a result, kappa values ranged from poor to substantial in the first session, showing a substantial difference between observers in intra-observer reliability. These values improved in the second session, with all observers showing a moderate to good agreement. From Sessions 2 to 3, kappa values remained similar or showed a slight decrease, which can likely be attributed to artifacts (e.g., excessive fat/skin obscuring the muscle, damaged breast muscle) in the new subset of photographs, which made scoring more difficult but represented more realistic examples. Considering the weighted kappa values, all observers had moderate to good agreement within themselves when considering the linear weighting. In contrast, all observers had good agreement when considering the quadratic weighting (Session 2 and 3). In the second session, the increase in kappa when using the quadratic weighing was larger for Observers 5 and 6, suggesting a larger difference in their scores (e.g., a score of 1 vs. 3 rather than 1 vs. 2). However, these were more similar across observers by the third session, suggesting only small differences in scores. 

### 3.2. Inter-Observer Reliability

The results of the inter-observer reliability assessment in the six rounds between all observers are shown in Table 2. In the initial round, there were no instances in which all observers agreed on the same score. Instead, in nearly half of the images, exactly three observers gave the same score. The agreement increased in subsequent rounds, with the best results observed in the third and fourth rounds, where ≥4 observers agreed on most scores. In these rounds, we also observed all six observers agreeing; however, this percentage was not higher than 32%, showing the difficulty in obtaining exact agreement on the scoring scale among six observers. This was also highlighted by the poor to fair kappa values for exact agreement and fair to moderate kappa values when using linear and quadratic weighing. In contrast, Kendall’s *W* was relatively high (>0.6), indicating good agreement for the ranking of the photographs. Similarly, Gwet’s agreement coefficient for ordinal data showed substantial to good agreement overall, especially when considering the quadratic weighing.

### 3.3. Prevalence of White Striping

**Figure 2:**
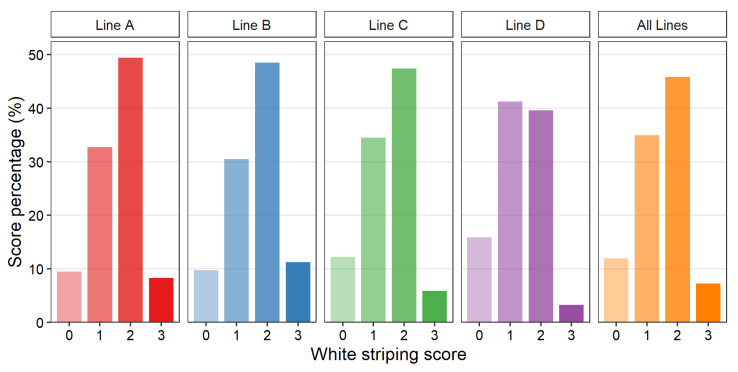
White striping severity score (Score 0–3 with 0 representing normal, 1 moderate, 2 severe, and 3 extreme) percentage within purebred turkey toms of each genetic line, A (*n* = 2839), B (*n* = 3728), C (*n* = 2034) or D (*n* = 3720) and within the entire studied population (*n* = 12,321).

The WS prevalence in the population studied is shown in Figure 2. Descriptive statistics for the recorded weight measures for each genetic line are shown in Table 3. The frequency of affected breasts (i.e., breasts scored as 1, 2, or 3) within the entire population was 88.1%. The four genetic lines showed similar prevalence, with Line A having the highest frequency at 90.5% and Line D the lowest frequency at 84.1%. Line D was the only line to show a higher proportion of lower scores (0 and 1) than higher scores (2 and 3), with the lowest frequency of breasts scored as extreme (3.3% as Score 3). In contrast, Line B had 11.3% of breasts scored as extreme (score of 3). Consequently, genetic line was found to be a significant factor when analyzing WS score (*p* < 0.05). Although it has the largest average slaughter weight and breast meat weight of the four genetic lines, Line D showed the lowest average WS score (2.30 ± 0.013, *p* < 0.05) compared to Line A (2.57 ± 0.015), Line B (2.61 ± 0.013), and Line C (2.47 ± 0.017). Average WS score of all lines significantly differed (*p* < 0.05) with the exception of lines A and B, which tended to have a more similar average WS score (*p* = 0.07). 

**Figure 3:**
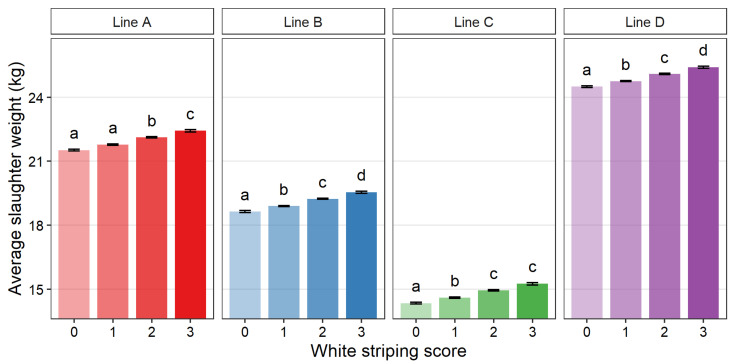
Least square means for slaughter weight (live weight of the bird 2 days prior to slaughter in kg) of purebred turkey toms for each white striping score (0–3) within each genetic line (A: *n* = 2834, B: *n* = 3715, C: *n* = 2032, D: *n* = 3701). Error bars show standard error. Means with different letters (a–d) within genetic line represent statistical significance (*p* < 0.05) as determined by Tukey’s HSD test.

**Figure 4:**
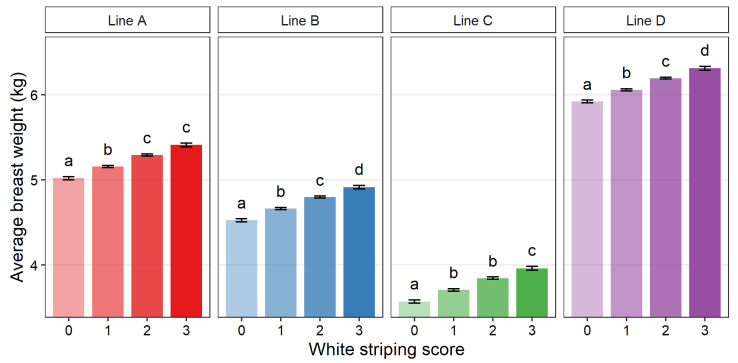
Least square means for breast weight (combined weight of *Pectoralis major* and *minor* muscles in kg) of purebred turkey toms for each white striping score (0–3) within each genetic line (A: *n* = 2830, B: *n* = 3677, C: *n* = 2031, D: *n* = 3652). Error bars show standard error. Means with different letters (a–d) within genetic line represent statistical significance (*p* < 0.05) as determined by Tukey’s HSD test.

**Figure 5:**
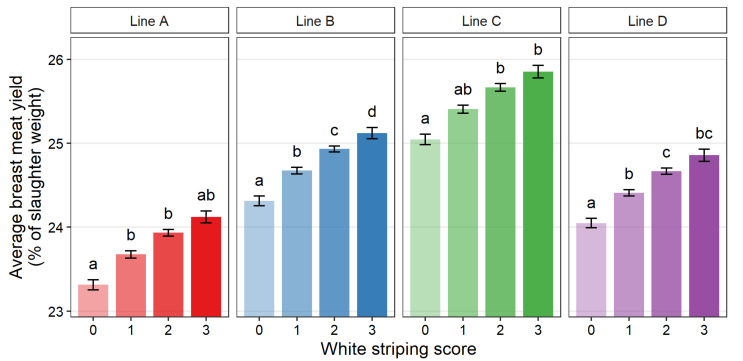
Least square means for breast meat yield (breast meat weight as a percentage of slaughter weight) of purebred turkey toms for each white striping score (0–3) within each genetic line (A: *n* = 2827, B: *n* = 3666, C: *n* = 2028, D: *n* = 3647). Error bars show standard error. Means with different letters (a–d) within genetic line represent statistical significance (*p* < 0.05) as determined by Tukey’s HSD test.

Figure 3, Figure 4 and Figure 5 show the average slaughter weight, breast weight, and BMY for each score within each line, respectively. Compared across lines, a similar trend was observed in that higher WS scores tended to be associated with higher average breast weight, slaughter weight, and BMY. For all lines, a WS score of 3 was associated with a significantly larger average weight measurement than score 0 (*p* > 0.05). The only exception was BMY of line A, which was numerically larger but not significantly different. The average percent increase between Score 0 and Score 3 was 4.6% for slaughter weight, 7.9% for breast weight, and 3.0% for BMY.

## 4. Discussion

The first objective of this study was to evaluate the reliability of a WS scoring scale for turkey breast muscles among multiple observers, followed by describing the prevalence and severity of WS in a turkey population consisting of birds from four genetic lines. Overall, we found that the scoring scale was reliable for multiple observers to score breasts for WS based on the cumulative information provided by the multiple coefficients estimated. Inter-observer reliability showed moderate to good agreement after completing three training sessions containing two rounds each. The inclusion of training is highly recommended for future studies attempting to score meat quality defects, such as WS, as the intra- and inter-observer reliability of the scoring system was improved after multiple sessions. Santos et al. [33] reported a weighted kappa of 0.85 for intra-reliability of WS in broiler chickens, which is similar to the results of the observers with the highest intra-observer reliability in the current study. It was not reported how many training sessions were required to reach this value in their study. To the best of the authors’ knowledge, no other studies have reported reliability measurements for WS in poultry. Reporting reliability measures is needed to evaluate and interpret data from such visual scoring systems. Observers in the current study appeared to stabilize their scoring and show the highest intra-observer reliability measures in Session 3 (Round 5 and 6), while the inter-observer reliability was strongest in Session 2 (Round 3 and 4). This is in line with research in other fields using discrete scoring systems to assess animal health. For example, D’Eath [46] reported that scoring reliability improved until the fifth scoring event. However, regular evaluation of the reliability of scoring systems and retraining is typically recommended.

This study suggests that the most difficulty in reaching agreement between observers was found between Score 1 and 2, which showed lower agreement coefficients. In contrast, the extremes of Score 0 and 3 were more easily agreed upon among all observers. Intermediate scores in discrete scales have previously been reported to have poorer reliability [45,46,47]. This difficulty in agreeing on Scores 1 and 2 could show a need for clearer definitions and examples, a broader scoring scale (fewer categories), or a reduction in the overall number of observers. More detailed scoring systems with more categories have more room for disagreement and require more training [46,47,48,49]. Depending on the purpose of the WS scoring, it could be recommended to reduce the scoring scale to combine Scores 1 and 2. However, merging scores is also considered to lead to a loss of granularity and should be suitable for the intended purpose of the assessment [46,47,48,49]. This seems relevant here, as Scores 1 and 2 were associated with differences in breast muscle weight, slaughter weight, and breast muscle yield in some of the genetic lines in the current study (Figure 3, Figure 4 and Figure 5). Ultimately, the development of a machine vision system capable of scoring breasts quantitatively, based on the number and thickness of the white striations, would be optimal; however, the development and use of such a program was not considered here.

It should be noted that this study used photographs to assess WS rather than live scoring due to practical constraints within the processing plant. Photographs or videos can be appropriate alternatives to live scoring [48,50]. Technology is increasingly implemented in processing plants to take advantage of automated methods (e.g., scoring of footpad lesions, grading broiler carcasses), which allow collection of larger datasets and benefits in terms of feasibility at high line-speeds [4]. However, disadvantages exist, and troubleshooting was required when initially setting up the camera system to ensure good quality photographs (e.g., adjustments for height, blur, and positioning). Feedback from observers showed that glare, positioning of breast outside of the area of focus, surface colour (striations were less noticeable with lighter coloured breast) or damage of the breast muscle (e.g., by trimming) could influence the scoring. These scenarios were more present in Session 3, which used less ideal photographs to mimic more realistic processing plant conditions. This could explain the slight dip in inter-observer reliability from Session 2 to 3, as observers adjusted to these conditions. These issues may be less of a problem when assessing breast muscle in-person. In-person training might be useful on a small subset of breasts for aligning scores between observers and an initial discussion of these scenarios, while time-consuming scoring of large samples sizes may be more appropriate via photographs. 

Compared to the results of this study, a lower prevalence of WS (61.3%) has previously been reported in turkeys using a 0–2 scoring system [25]. A potential factor worth considering in future studies is the type of birds used, since pedigree stock, which was used in the present study, tends to be larger and may show differing levels of WS compared to commercial birds that are typically a hybrid cross. The WS prevalence of the studied population was similar, if not slightly higher than that previously published in broiler chickens, ranging between 56–87% in heavy strains [10,22,23,24]. A case has been made for WS being an emerging problem in broiler chickens over the past years and that this issue may also be present in turkeys.

Fast-growing broiler chickens and turkeys have been shown to have increased severity of WS [10,12,13,31]. The same finding was also observed within each line in the present study. However, this was not the case when comparing WS and average body weight between the four studied lines (i.e., the largest line, Line D, showed the lowest WS severity). Increases in breast weight between normal breasts and breasts affected by WS in broilers range from 16% to 25% [12,20], and an increase of 20% was observed in turkeys [13]. These reported differences in breast muscle weight between normal and affected breasts were higher than those observed in the present study between normal and extreme breasts (average of 7.9% increase). However, it should be noted that the weight used in the present study was the total weight of both *Pectoralis major* and *minor* muscles, which may influence the correlation between breast weight and WS. Significant increases in both slaughter weight and BMY between normal and extreme breast fillets were also observed in this study (average of 4.6% and 3.0%, respectively). This negative association between larger birds (i.e., one of the major economic goals of poultry breeding), and increases in muscle myopathies, such as WS, may pose an issue for breeders that could be managed through a selection index with appropriate economic weights. 

It is noteworthy that the four lines studied had differing average slaughter ages depending on the birds’ size. The largest two lines, A and D, had an average slaughter age of 148.1 days and 143.8 days, respectively. The smaller two lines, B and C, had an average slaughter age of 153.7 days and 156.2 days. We do not believe this is a significant factor in the prevalence results as the range between the highest and lowest slaughter ages is minimal; however, it should be considered as age has been shown to affect the severity of WS [51].

## 5. Conclusions

The reliability of a WS scoring system for turkey breast muscles with multiple observers was developed and assessed. Later, the prevalence of WS in a large population of purebred turkey toms was determined. Overall, we report that the scoring system showed moderate to good reliability within and between the observers and that reliability improved after multiple training sessions. The prevalence of WS varied across the studied turkey lines and was 88.1% on average, with the majority of scores being moderate-severe (Scores 1 and 2). This highlights that this breast muscle myopathy is quite prevalent in turkeys and should continue to be investigated due to its negative effect on consumer preference and composition of the meat. In general, WS severity was found to be associated with higher slaughter weight, breast weight, and BMY, suggesting negative correlations that will have to be dealt with using a balanced selection index to reduce the presence of WS while continuing to maintain an increase in these economically significant traits.

## Figures and Tables

**Table 1:** Intra-observer reliability coefficients of white striping scoring of turkey breast muscle (0–3 scale) to assess agreement within 6 observers over multiple training sessions. The 95% confidence interval for kappa, linear weighted kappa, and quadratic weighted kappa are given below the coefficients in brackets.

	Observer 1	Observer 2	Observer 3	Observer 4	Observer 5	Observer 6	Average
**Session 1 (round 1 and 2) ^1^**							
Exact agreement (%)	38	74	68	78	62	74	66
Spearman correlation	0.48 **	0.68 ***	0.69 ***	0.79 ***	0.63 ***	0.81 ***	0.68
Kappa	0.12 (–0.05–0.29)	0.51 (0.28–0.73)	0.43 (0.21–0.65)	0.64 (0.45–0.82)	0.41 (0.21–0.61)	0.62 (0.44–0.80)	0.45
Linear weighted kappa	0.20 (0.06–0.34)	0.58 (0.37–0.78)	0.54 (0.35–0.73)	0.70 (0.54–0.86)	0.51 (0.33–0.69)	0.72 (0.58–0.86)	0.54
Quadratic weighted kappa	0.31 (0.17–0.46)	0.68 (0.50–0.86)	0.68 (0.53–0.83)	0.78 (0.66–0.91)	0.62 (0.45–0.80)	0.82 (0.72–0.92)	0.65
**Session 2 (round 3 and 4) ^1^**							
Exact agreement (%)	80	86	88	74	68	72	78
Spearman correlation	0.73 ***	0.87 ***	0.86 ***	0.67 ***	0.70 ***	0.74 ***	0.76
Kappa	0.62 (0.41–0.83)	0.77 (0.61–0.93)	0.80 (0.64–0.95)	0.53 (0.31–0.75)	0.47 (0.27–0.68)	0.56 (0.36–0.76)	0.63
Linear weighted kappa	0.66 (0.46–0.85)	0.82 (0.68–0.95)	0.83 (0.70–0.96)	0.59 (0.39–0.79)	0.57 (0.40–0.75)	0.65 (0.49–0.82)	0.69
Quadratic weighted kappa	0.71 (0.50–0.91)	0.87 (0.77–0.97)	0.88 (0.78–0.98)	0.67 (0.49–0.84)	0.70 (0.56–0.83)	0.76 (0.63–0.89)	0.76
**Session 3 (round 5 and 6) ^2^**							
Exact agreement (%)	80	86	88	76	80	76	81
Spearman correlation	0.65 ***	0.88 ***	0.86 ***	0.65 ***	0.81 ***	0.71 ***	0.76
Kappa	0.59 (0.37–0.81)	0.80 (0.66–0.94)	0.80 (0.65–0.95)	0.52 (0.29–0.76)	0.69 (0.51–0.86)	0.58 (0.38–0.78)	0.66
Linear weighted kappa	0.62 (0.41–0.83)	0.84 (0.72–0.96)	0.83 (0.69–0.96)	0.57 (0.36–0.79)	0.75 (0.61–0.90)	0.63 (0.46–0.81)	0.71
Quadratic weighted kappa	0.67 (0.47–0.88)	0.88 (0.77–0.99)	0.87 (0.76–0.97)	0.64 (0.45–0.83)	0.83 (0.72–0.94)	0.71 (0.55–0.86)	0.77

^1^ Sessions 1 and 2 were conducted with the same set of 50 photographs; ^2^ Session 3 was conducted with a new set of 50 photographs, ** *p* < 0.01, *** *p* < 0.001.

**Table 2:** Inter-observer reliability coefficients of white striping scoring of turkey breast muscle (0–3 scale) to assess agreement between 6 observers over multiple training sessions. The 95% confidence interval for kappa, linear weighted kappa, quadratic weighted kappa, and Gwet’s AC_2_ are given below the coefficients in brackets.

	Session 1 ^1^		Session 2 ^1^		Session 3 ^2^	
	Round 1	Round 2	Round 3	Round 4	Round 5	Round 6
Percentage agreement (%) ^3^						
All 6 observers agree	0	6	32	22	12	10
5 observers agree	24	24	28	36	32	28
4 observers agree	24	46	36	26	26	40
3 observers agree	48	24	4	16	26	22
2 observers agree	4	0	0	0	4	0
Exact agreement						
Kappa	0.17 (0.10−0.25)	0.27 (0.20–0.34)	0.33 (0.26–0.39)	0.29 (0.22–0.36)	0.21 (0.15–0.28)	0.24 (0.16–0.31)
Kendall’s *W*	0.61 ***	0.68 ***	0.74 ***	0.70 ***	0.61 ***	0.66 ***
Linear weighting						
**Linear weighted kappa**	0.32 (0.20–0.44)	0.45 (0.36–0.54)	0.52 (0.45–0.59)	0.48 (0.39–0.56)	0.38 (0.28–0.47)	0.41 (0.31–0.51)
Gwet’s AC_2_						
Overall	0.54 (0.28–0.81)	0.65 (0.56–0.74)	0.79 (0.69–0.89)	0.76 (0.66–0.87)	0.67 (0.58–0.77)	0.68 (0.58–0.79)
Score 0	0.88 (0.80–0.96)	0.86 (0.77–0.94)	0.93 (0.88–0.99)	0.96 (0.91–1.00)	0.88 (0.78–0.97)	0.89 (0.80–0.97)
Score 1	0.47 (0.28–0.66)	0.51 (0.32–0.70)	0.72 (0.59–0.84)	0.70 (0.51–0.89)	0.57 (0.41–0.72)	0.60 (0.43–0.78)
Score 2	0.49 (0.26–0.72)	0.58 (0.45–0.72)	0.68 (0.53–0.84)	0.61 (0.46–0.77)	0.58 (0.44–0.73)	0.56 (0.38–0.73)
Score 3	0.81 (0.58–1.00)	0.92 (0.86–0.98)	0.93 (0.87–0.99)	0.89 (0.81–0.96)	0.91 (0.84–0.98)	0.89 (0.81–0.97)
Quadratic weighting						
**Quadratic weighted kappa**	0.32 (0.20–0.44)	0.45 (0.36–0.54)	0.52 (0.45−0.59)	0.48 (0.39–0.56)	0.38 (0.28–0.47)	0.41 (0.31–0.51)
Gwet’s AC_2_						
Overall	0.74 (0.53–0.95)	0.83 (0.77–0.88)	0.91 (0.85–0.96)	0.89 (0.83–0.95)	0.83 (0.76–0.90)	0.85 (0.78–0.92)
Score 0	0.88 (0.80–0.96)	0.86 (0.77–0.95)	0.93 (0.88–0.99)	0.96 (0.91–1.00)	0.88 (0.78–0.97)	0.89 (0.80–0.97)
Score 1	0.47 (0.28–0.66)	0.51 (0.32–0.70)	0.72 (0.59–0.84)	0.70 (0.51–0.89)	0.57 (0.42–0.72)	0.60 (0.43–0.78)
Score 2	0.49 (0.26–0.72)	0.58 (0.45–0.72)	0.68 (0.53–0.84)	0.61 (0.46–0.77)	0.58 (0.44–0.73)	0.56 (0.38–0.73)
Score 3	0.81 (0.58–1.00)	0.92 (0.86–0.98)	0.93 (0.87–0.99)	0.89 (0.81–0.96)	0.91 (0.84–0.98)	0.89 (0.81–0.97)

^1^ Sessions 1 and 2 were conducted with the same set of 50 photographs; ^2^ Session 3 was conducted with a new set of 50 photographs; ^3^ Percentage agreement by each exact number of observers; *** *p* < 0.001.

**Table 3:** Descriptive statistics (count, mean, and standard deviation) for white striping score, slaughter weight, breast weight, and breast meat yield (BMY) for the four genetic lines of turkeys studied.

Genetic Line	White Striping Score (0–3)			Slaughter Weight (kg)			Breast Weight (kg)			BMY (% BW)		
	N	Mean	SD	N	Mean	SD	N	Mean	SD	N	Mean	SD
**A**	2839	2.57	0.776	2834	21.98	1.548	2830	5.23	0.583	2827	23.81	1.966
**B**	3728	2.61	0.81	3715	19.11	1.286	3677	4.74	0.499	3666	24.81	1.789
**C**	2034	2.47	0.782	2032	14.77	1.005	2031	3.77	0.394	2028	25.51	1.704
**D**	3720	2.30	0.771	3701	24.87	1.801	3652	6.10	0.757	3647	24.47	2.108

## Data Availability

Data available from the corresponding author upon reasonable request.
